# Imaging of Embryonic and Fetal Brain Development Using MRI Microscopy: Achieving High Spatial Resolution

**DOI:** 10.3390/medsci14020219

**Published:** 2026-04-28

**Authors:** Dan Boitor, Alexandru Farcasanu, Simion Simon, Daniel Muresan, Ioana Cristina Rotar, Mihai Surcel, Mihaela Oancea

**Affiliations:** 1 Department of Obstetrics and Gynecology, “Iuliu Hatieganu” University of Medicine and Pharmacy, 400012 Cluj-Napoca, Romania; dan.boitor@umfcluj.ro (D.B.); daniel.muresan@umfcluj.ro (D.M.); cristina.rotar@umfcluj.ro (I.C.R.); mihai.surcel@umfcluj.ro (M.S.); mihaela.oancea@umfcluj.ro (M.O.); 2 INSPIRE Platform, Babes-Bolyai University, 400347 Cluj-Napoca, Romania; 3 Amethyst Radiotherapy Center Cluj, 407280 Florești, Romania

**Keywords:** MRI microscopy, micro-MRI, high resolution, high magnetic field, brain development

## Abstract

The visualization of embryonic and fetal brain development at mesoscopic resolution represents a critical frontier in developmental neuroscience. This review presents advances in high-field magnetic resonance imaging (HF-MRI) that achieve unprecedented spatial resolution in ex vivo human embryonic and fetal brain specimens. This mesoscopic imaging capability bridges the gap between conventional clinical MRI and histological microscopy, enabling three-dimensional visualization of transient developmental structures including cortical lamination, ganglionic eminences, and emerging white matter pathways. We review the technical foundations of HF-MRI, present methodological advances that enable mesoscopic resolution, demonstrate applications across gestation, and discuss validation through histological correlation. The integration of multimodal imaging approaches—including T1-weighted, T2-weighted, T2*-weighted, diffusion tensor imaging, and quantitative relaxometry—provides comprehensive characterization of tissue microstructure and connectivity during critical periods of neurodevelopment. These advances offer transformative potential for understanding normal brain development, identifying early markers of neurodevelopmental disorders, and establishing high-resolution atlases of human prenatal neuroanatomy.

## 1. Introduction

Understanding human brain development from embryonic stages through the fetal period is fundamental to developmental neuroscience, pediatric neurology, and the study of neurodevelopmental disorders. The human brain undergoes rapid and complex structural changes during prenatal development, including neurogenesis, neuronal migration, axonal pathfinding, synaptogenesis, and the formation of transient developmental zones such as the subplate, cortical plate, and ganglionic eminences [1,2]. These processes occur at spatial scales ranging from millimeters to micrometers, presenting significant challenges for non-invasive imaging.

Conventional clinical magnetic resonance imaging (MRI) at field strengths of 1.5 to 3 Tesla (T) has provided valuable insights into fetal brain development in utero, enabling visualization of major anatomical structures, cortical folding patterns, and gross volumetric changes [3,4]. However, clinical MRI is fundamentally limited by spatial resolution (typically 0.5–1.0 mm isotropic), motion artifacts from fetal movement, and signal-to-noise ratio (SNR) constraints that preclude visualization of anatomical features [5,6]. These limitations are particularly problematic during early gestational stages when the brain is small and rapidly changing, and when transient developmental structures cannot be resolved at conventional resolutions.

Ex vivo high field MRI (HF-MRI) at 4 T and above offers a transformative approach to studying prenatal brain development by dramatically increasing spatial resolution while maintaining 3D coverage and tissue contrast [7,8,9]. Compared to conventional clinical MRI at 1.5–3 T, HF-MRI provides order-of-magnitude improvements in spatial resolution, enabling visualization of structures invisible at clinical field strengths [3,5]. Recent advances in hardware design, pulse sequence optimization, and image reconstruction have pushed the boundaries of achievable resolution into the mesoscopic domain, with studies reporting resolutions of 100 micrometers (µm) at 11.7 T [1,6], 18–20 µm at 9.4 T [10,11], and 27 µm at 7.04 T [12]. These achievements represent a convergence toward true MR microscopy of human embryonic and fetal brain tissue, enabling visualization of laminar structures, nuclear groups, and fiber pathways that were previously accessible only through destructive histological sectioning.

The aim of this review is to present recent breakthroughs in HF-MRI that achieve outstanding spatial resolution in ex vivo human embryonic and fetal brain specimens. We use “high resolution” as a relative descriptor, always accompanied by the specific value, indicating substantial improvement over clinical fetal brain MRI. We adopt standardized resolution terminology based on quantitative ranges (Table 1).

## 2. Methods and Scope

### 2.1. Search Strategy and Data Sources

This review was conducted following established methodological frameworks for narrative reviews. A comprehensive literature search was performed across core articles in the Web of Science database. The search strategy combined keywords related to -high field MRI technology with terms describing fetal, embryonic, and prenatal brain imaging applications (“micro-MRI” OR “MRI microscopy” OR “high field”) AND (“brain” OR “cerebrum” OR “cerebral”) AND (“fetal” OR “embryonic”) AND (“high resolution”) AND (“development”). A total of 141 studies were retrieved.

### 2.2. Study Selection Criteria

Studies were included if they: (1) utilized MRI at field strengths of 4 T or higher, (2) focused on embryonic, fetal, or early developmental brain imaging, (3) explicitly reported spatial resolution, and (4) provided sufficient technical detail to enable quantitative comparison of acquisition parameters and image quality metrics. Both human and animal model studies were included to capture the full scope of HF-MRI applications in developmental neuroscience. Supplementary filters were applied: publications in English and those released after the year 2000. A total of 46 unique papers fulfilling the inclusion criteria were identified and ranked based on their direct relevance to high-field MRI applications in fetal and embryonic brain imaging. These articles were then manually reviewed to exclude those that were mistakenly selected or focused on unrelated methodologies, where MRI was only used as a supporting method without significant relevance to our review. We explicitly excluded: (1) in vivo fetal MRI studies, which operate under fundamentally different constraints (motion, scan time limitations, safety considerations) that preclude direct technical comparison; (2) lower-field studies (<4 T), which represent a distinct technical regime with different signal-to-noise and contrast mechanisms; (3) studies reporting only qualitative findings without quantitative resolution specifications; and (4) conference abstracts or non-peer-reviewed reports lacking sufficient methodological detail. For in-depth comparison analysis, six core studies were selected through systematic criteria to ensure meaningful technical comparisons. These papers were analyzed in detail, with systematic extraction of information on field strength, imaging modalities, study design, sample characteristics, and key findings. This approach ensures that comparisons are made between studies employing similar physical principles (ex vivo tissue imaging at high field) and addressing comparable scientific questions (anatomical characterization of brain structure). Table 2 summarizes key studies achieving high resolution in human embryonic and fetal brain ex vivo imaging.

We acknowledge that this focused selection strategy, while enabling rigorous quantitative comparison, may overlook methodological innovations reported in the broader fetal brain imaging literature, including specialized techniques for motion correction, accelerated acquisition, or novel contrast mechanisms. A comprehensive systematic review of all fetal brain MRI methodologies falls outside the scope of this technical comparison but would provide valuable context for future work.

## 3. Technical Foundations of High-Field MRI

### 3.1. Field Strength and Signal-to-Noise Ratio

The fundamental advantage of high-field MRI lies in the relationship between magnetic field strength and signal-to-noise ratio (SNR). SNR increases approximately linearly with field strength, providing the foundation for higher spatial resolution imaging [14]. At 11.7 T, the theoretical SNR gain relative to 3 T clinical systems is approximately 3.7-fold, although practical gains are moderated by factors including increased T1 relaxation times, shortened T2 relaxation times, and technical challenges related to radiofrequency (RF) field inhomogeneity and specific absorption rate (SAR) constraints [15]. For ex vivo specimens, several factors further enhance SNR compared to in vivo imaging. First, the absence of physiological motion eliminates the need for rapid acquisition sequences, permitting long scan times (often 12–24 h) with extensive signal averaging [12,16]. Second, specimens can be positioned optimally within small-bore, high-performance gradient systems that provide superior field homogeneity and gradient strength [10]. Third, specialized RF coils can be designed to match specimen geometry, maximizing filling factor and SNR [17].

### 3.2. Hardware Considerations

Achieving high spatial resolution requires specialized hardware beyond standard clinical MRI systems. Key components include the following: (1) High-performance gradient systems: Modern preclinical MRI systems at 7 T and above typically feature gradient strengths of 400–1000 mT/m, compared to 40–80 mT/m in clinical systems [18]. These strong gradients enable short echo times and high spatial encoding efficiency [19]; (2) Optimized RF coils: Custom-designed radiofrequency coils matched to specimen size are critical for maximizing SNR. For embryonic and early fetal specimens (crown-rump length 20–100 mm), small solenoid or surface coils provide superior performance compared to larger volume coils [12]; (3) Temperature control and specimen preparation: Specimens are typically immersed in perfluoropolyether or formalin-based solutions that minimize susceptibility artifacts while preserving tissue structure [20]. Temperature stabilization prevents thermal drift that could degrade image quality during long acquisitions.

## 4. Methodological Advances Enabling Mesoscopic Spatial Resolution

The choice and optimization of pulse sequences is critical for achieving mesoscopic resolution. Recent studies achieving high resolution have employed several approaches: three-dimensional gradient echo sequences [12]; T2-wi spin echo sequences (three-dimensional rapid acquisition with relaxation enhancement (RARE), and turbo spin echo (TSE)) [2,21]; compressed sensing and parallel imaging [10].

To achieve high spatial resolution in human embryonic and fetal brain imaging, a FISP-3D protocol was used at 7.04 T, with T1-weighted images providing hyperintense signals that identified ganglionic eminences and other developing structures [12]. Importantly, this resolution approaches the scale of individual cortical layers (which range from approximately 50–500 µm in thickness during fetal development) and is sufficient to resolve major axonal pathways and nuclear boundaries [2]. Parallel developments at 11.7 T have established complementary capabilities at slightly lower but still mesoscopic resolution. The premature Human Connectome Project (p-HCP) has developed multimodal imaging protocols at 11.7 T that achieve approximately 100 µm isotropic resolution for anatomical imaging and 200 µm for diffusion-weighted imaging across the full span of prenatal development from 20 to 41 gestational weeks [1,6]. The 11.7 T approach offers several advantages despite the slightly lower spatial resolution compared to published studies at 7.04 T. First, the higher field strength provides superior SNR that can be traded for reduced scan time or improved contrast-to-noise ratio [22]. Second, the 11.7 T systems enable whole-hemisphere or whole-brain imaging with consistent resolution, whereas the highest resolution studies often focus on smaller regions of interest [6]. Third, the 11.7 T protocols integrate multiple contrasts—T1, T2, T2*, and diffusion-weighted imaging—within a unified framework, providing comprehensive tissue characterization [1,6].

Studies at 4.7 T have achieved resolutions of 130–200 µm in fetal brain specimens [2,13], while 7.04 T systems have demonstrated resolutions of 18–27 µm [10,11,12], and 9.4–11.7 T systems have achieved 10–100 µm depending on specimen size and acquisition time [1,6,10]. The relationship between field strength and achievable resolution is not strictly linear due to competing factors. Higher fields provide greater SNR but also face challenges including increased susceptibility artifacts, RF field inhomogeneity (*B*_1_+ inhomogeneity), and SAR constraints [23]. For ex vivo specimens, SAR is less limiting than in vivo, but *B*_1_+ inhomogeneity and susceptibility effects remain significant concerns that require careful shimming and pulse sequence design [24].

## 5. Applications Across Developmental Stages

### 5.1. Early Embryonic Period (9–12 Gestational Weeks)

The early embryonic period represents a critical window when major brain structures are first forming. At 9–12 gestational weeks, the brain is characterized by the presence of large germinal zones, the emergence of the cortical plate, and the initial formation of ganglionic eminences that will give rise to basal ganglia and interneurons [25]. At 7.04 T, Boitor-Borza et al. demonstrated clear delineation of the lateral, medial, and caudal ganglionic eminences in specimens from 9 to 14 gestational weeks, with the ability to track morphological changes as these structures evolve [12]. Studies at 9.4 T achieving 10–12 µm resolution have similarly demonstrated the ability to visualize brain structures in human embryos, with microstructures well delineated and comparable to optical microscopy images [10]. At these resolutions, individual layers within the developing cerebral wall can be distinguished, including the ventricular zone, intermediate zone, and cortical plate [11].

### 5.2. Mid-Fetal Period (13–22 Gestational Weeks)

During this period, the cerebral wall exhibits complex laminar organization with up to six distinct layers visible on high-resolution MRI [2]. Diffusion tensor imaging (DTI) at 11.7 T with 200–400 µm resolution has provided complementary information about tissue microstructure during this period. Huang et al. (2013) demonstrated that fractional anisotropy (FA) values in the developing cortex correlate with the degree of radial organization, with high FA in regions of organized radial glia and lower FA in regions undergoing tangential migration [13].

### 5.3. Late Fetal Period (23–41 Gestational Weeks)

The p-HCP dataset provides mesoscopic resolution (100 µm) anatomical and diffusion imaging from 20 to 41 gestational weeks, enabling quantitative analysis of cortical surface area, thickness, and folding patterns [1,6]. At 100 µm resolution, individual gyri and sulci can be traced with precision, and the timing of sulcal formation can be documented with gestational-week accuracy [26]. Quantitative relaxometry at 11.7 T (T1, T2, and T2* mapping at 200 µm resolution) provides markers of tissue maturation that correlate with myelination and cellular density changes [6]. T1 values decrease, and T2 values shorten as white matter matures, providing quantitative biomarkers of developmental progression that complement anatomical imaging [27].

### 5.4. Comparative Developmental Trajectories

Integration of data across developmental stages enables characterization of growth trajectories and identification of critical developmental milestones. Studies using motion-corrected in vivo MRI at 1.5 T have demonstrated sex-specific differences in brain growth trajectories beginning in mid-gestation, with males showing larger white matter volumes and females showing greater occipital lobe curvature [28]. These findings, obtained at 500 µm resolution, suggest that even lower resolution ex vivo studies may reveal additional subtle differences in cortical organization and connectivity. The ability to image the same structures across a wide gestational age range with consistent methodology enables the construction of four-dimensional (3D + time) atlases that capture the dynamic nature of brain development [29]. Such atlases provide normative references for identifying deviations from typical development and for planning clinical imaging studies [30].

## 6. Multimodal Imaging and Tissue Characterization

### 6.1. T1-Weighted and T2-Weighted Imaging

The combination of T1-weighted and T2-weighted imaging provides complementary information about tissue composition and developmental state. T1-weighted images are sensitive to tissue water content and macromolecular concentration, with shorter T1 values (brighter signal) in regions with higher lipid content or lower water content [31]. Studies employing both contrasts have demonstrated that certain structures require specific contrasts for optimal visualization. Kunieda et al. (2024) found that detailed structures within the cerebral cortex and accessory nerves were not delineated in T1-weighted images and required T2*-weighted gradient echo imaging for visualization [11]. This highlights the importance of multimodal approaches that employ multiple contrasts to comprehensively characterize tissue properties.

### 6.2. Diffusion MRI and Connectivity Mapping

Diffusion MRI, including diffusion tensor imaging (DTI) and high angular resolution diffusion imaging (HARDI), provides unique information about tissue microstructure and connectivity that complements anatomical imaging [32]. The integration of DTI with histological analysis confirmed that FA patterns correspond to specific cellular architectures visible in histological sections [13]. High angular resolution diffusion imaging at 11.7 T enables tractography-based reconstruction of developing white matter pathways [1,6]. At 200 µm resolution, major commissural, projection, and association pathways can be traced with precision, enabling quantitative analysis of pathway development across gestational age [33]. Studies in animal models have demonstrated that diffusion MRI can detect subtle alterations in connectivity resulting from prenatal insults, suggesting potential applications for identifying early markers of neurodevelopmental disorders [34].

The combination of multiple quantitative parameters enables tissue classification and segmentation based on objective criteria rather than subjective intensity thresholds [35,36,37]. This is particularly valuable for automated analysis of large datasets and for comparing findings across studies and imaging platforms [38].

## 7. Validation Through Histological Correlation

### 7.1. Biological and Cellular Basis of MRI Contrasts in Fetal Brain Imaging

A critical requirement for establishing MRI as a valid tool for studying brain development is demonstrating correspondence between MRI-defined structures and histologically verified anatomy. Multiple studies have performed systematic comparisons between high-resolution MRI and histological sections from the same specimens [2,11,13,21], demonstrating strong correspondence between MRI-defined structures and histologically identified features.

The different MRI contrasts employed reflect distinct biological and cellular tissue properties that evolve during fetal brain development. Quantitative mapping of relaxation parameters (T1, T2, and T2*) provides objective biomarkers of tissue composition and maturation [6]. These parameters are sensitive to water content, macromolecular concentration, iron content, and microstructural organization, all of which change systematically during brain development [35]. T1 values in white matter decrease from approximately 2000–2500 ms at 20 gestational weeks to 1500–2000 ms at term, reflecting decreasing water content and increasing macromolecular concentration associated with myelination [15,27]. T2-weighted images provide strong anatomical contrast, as they are sensitive to tissue water content and microstructural organization, with longer T2 values (brighter signal) in regions with higher water content or less organized structure, such as gray matter [2]. Both T1 and T2 values decrease during brain development. T2*-weighted imaging and quantitative susceptibility mapping (QSM) detect iron accumulation in deep gray matter structures, particularly the globus pallidus, after 30 gestational weeks, reflecting early iron deposition associated with neuronal maturation and serving as markers of developmental progression [16,17,36]. The germinal matrix, a highly vascularized proliferative region, shows increased susceptibility signal in T2* and QSM images due to its high vascularity and cellular density [28,29]. Diffusion tensor imaging (DTI) at 100 μm resolution enables tractography of major white matter pathways and quantification of microstructural development through fractional anisotropy measurements, which increase with white matter maturation as axons become more organized; for example, the subplate shows lower fractional anisotropy (0.15–0.25) compared to the intermediate zone (0.30–0.45), reflecting its loose organization and waiting synaptic connections [18,19]. Together, these complementary contrasts provide comprehensive characterization of tissue microstructure, composition, and developmental maturation, with each modality sensitive to specific cellular and molecular properties including water content, lipid composition, iron concentration, vascularity, and axonal organization [15,16,17,18,19,29,37]. This is particularly valuable for automated analysis of large datasets and for comparing findings across studies and imaging platforms [38]. Continued integration of MRI with histology, electron microscopy, and molecular analysis is needed to fully understand the biological basis of MRI contrast [13].

### 7.2. Limitations of Histological Validation

While histological correlation provides essential validation, several limitations must be acknowledged. First, histological processing involves tissue deformation, shrinkage, and potential artifacts that can alter the appearance and dimensions of structures [39]. Second, histological sectioning is typically performed in a single plane, whereas MRI provides true three-dimensional data, making precise registration challenging [40]. Third, the destructive nature of histology means that only a subset of specimens can be processed for validation, and once sectioned, the specimen cannot be re-imaged [20]. Despite these limitations, the strong correspondence between MRI and histology across multiple studies and developmental stages provides confidence that MRI-defined structures reflect genuine anatomical features [2,11,13]. The ability of MRI to provide non-destructive, three-dimensional visualization represents a significant advantage over histology for creating comprehensive developmental atlases [41].

### 7.3. Functional Validation Through Gene Expression

An emerging approach to validation involves correlating MRI findings with gene expression patterns. The Allen Brain Atlas and similar resources provide comprehensive gene expression data for the developing human brain, enabling correlation between MRI-defined regions and molecular signatures [13]. Huang et al. integrated DTI with gene expression analysis from the Allen Brain Atlas, demonstrating that regions with high FA values in DTI correspond to areas with high expression of genes involved in radial glia function and neuronal migration, while regions with lower FA show enrichment for genes involved in tangential migration and interneuron development [13]. This molecular validation provides mechanistic insights into the cellular processes underlying MRI contrast and supports the biological relevance of MRI-defined features [13].

## 8. Comparative Analysis of Field Strengths and Resolution

### 8.1. Resolution Scaling Across Field Strengths

**Figure 1:**
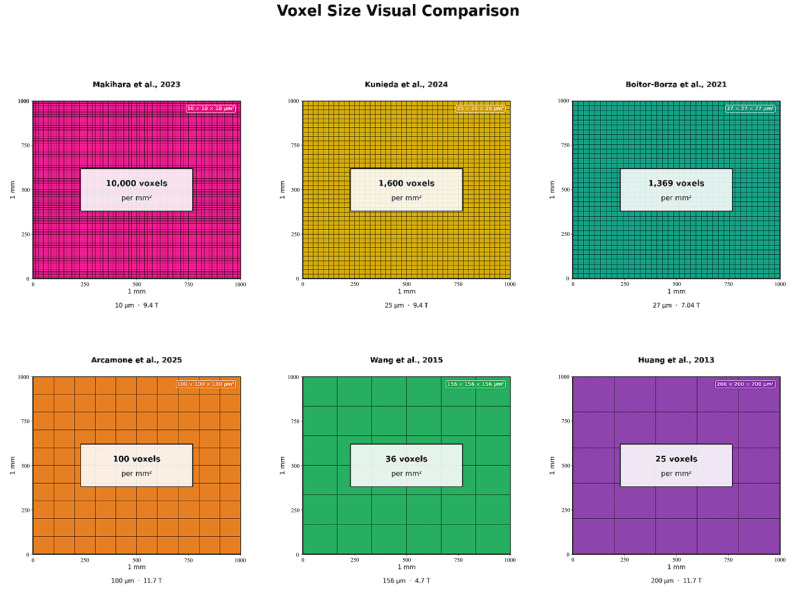
Voxel size visual comparison across selected studies. Each panel represents a 1 mm × 1 mm cross-section tiled with voxels at the study’s reported in-plane resolution. The voxel count per mm^2^ directly illustrates information-density differences between acquisitions [2,6,10,11,12,13].

**Figure 2:**
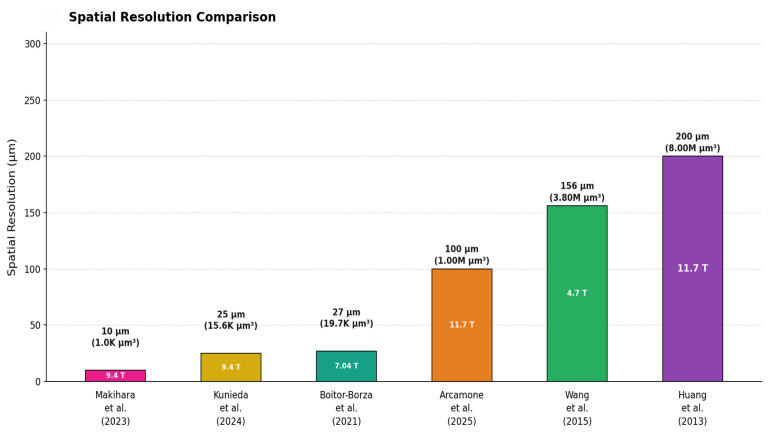
Resolution comparison across selected studies, ordered from finest to coarsest. Bar height = representative isotropic voxel edge length (μm); annotation shows voxel volume (μm^3^). Field strength (T) is indicated [2,6,10,11,12,13].

Comparative analysis across field strengths reveals systematic relationships between magnetic field, resolution, specimen characteristics, and scan time. Figure 1 shows a very compelling visual comparison of voxel sizes across selected studies. Resolution scales and key milestones in spatial resolution across different field strengths are clearly illustrated in Figure 2.

Several patterns are revealed. First, the highest spatial resolutions (10–27 µm) have been achieved at field strengths of 7.04–9.4 T in small specimens (embryos and early fetuses) [10,11,12]. Second, studies at 11.7 T have focused on achieving mesoscopic and sub-millimetric resolution (100–200 µm) across larger specimens and wider gestational age ranges [1,6,13]. Third, there is a trade-off between resolution and coverage, with the highest resolution studies typically imaging smaller regions or specimens [10,12]. Langner et al. reported a remarkable spatial resolution of 20 µm (in-plane) when investigating the upper extremity ossification and muscles in specimens of 8–12 gestational weeks, operating at 7.1 T [21]. As the results do not apply to the brain of the specimens, this reference was not taken into consideration in our study.

### 8.2. SNR Efficiency Across Platforms

The efficiency of different field strengths for achieving specific resolutions can be assessed by comparing SNR per unit time. While direct comparisons are complicated by differences in hardware, pulse sequences, and specimen preparation, some general principles emerge. Higher field strengths provide greater SNR per unit time, but this advantage is partially offset by increased T1 relaxation times (which reduce steady-state signal in gradient echo sequences) and shortened T2* relaxation times (which increase susceptibility artifacts and reduce signal in gradient echo sequences) [42]. For ex vivo specimens with long T1 values, the SNR advantage of higher fields is substantial, supporting the use of 11.7 T for comprehensive multimodal imaging [1,6]. However, the 27 µm resolution achieved at 7.04 T demonstrates that exceptional resolution can be obtained at somewhat lower fields when imaging small specimens with optimized hardware and extended acquisition times [12]. This suggests that field strength is not the sole determinant of achievable resolution; hardware quality, pulse sequence optimization, and acquisition time are equally important factors [43].

### 8.3. Practical Considerations for Study Design

The choice of field strength and target resolution should be guided by the specific scientific questions and practical constraints of each study. For comprehensive imaging across wide gestational age ranges, 11.7 T systems with 100–200 µm resolution provide an excellent balance of resolution, coverage, and multimodal capability [1,6]. For detailed studies of specific structures in early embryonic specimens, 7.04–9.4 T systems with 10–30 µm resolution enable visualization approaching histological detail [10,11,12]. Balancing resolution, coverage, and throughput requires careful consideration of study goals and available resources [44].

## 9. Discussion

### 9.1. Scientific Significance

The achievement of high spatial resolution in MRI of human embryonic and fetal brain represents a transformative advance in developmental neuroimaging. It approaches the scale of individual cortical layers, major axonal bundles, and small nuclear groups, enabling visualization of structures that were previously accessible only through destructive histological methods [12]. The ability to image these structures in three dimensions, with multiple contrasts in intact specimens, provides unprecedented opportunities for understanding normal brain development and identifying early markers of neurodevelopmental disorders [45]. The integration of high-resolution anatomical imaging with diffusion MRI, quantitative relaxometry, and molecular data creates a comprehensive framework for characterizing brain development across multiple scales [1,6,13]. This multimodal approach enables investigation of relationships between macroscopic anatomy, tissue microstructure, connectivity, and gene expression, providing mechanistic insights into developmental processes [13].

### 9.2. Comparison with Alternative Imaging Modalities

Ultra-high field MRI occupies a unique niche in the landscape of developmental neuroimaging techniques. Compared to conventional clinical MRI at 1.5–3 T, UHF-MRI provides order-of-magnitude improvements in spatial resolution, enabling visualization of structures invisible at clinical field strengths [3,5]. MRI provides a unique combination of three-dimensional coverage, soft tissue contrast, and non-destructive imaging that is particularly well-suited for creating comprehensive developmental atlases [41]. Compared to histology, MRI provides non-destructive three-dimensional imaging with multiple contrasts, although histology retains advantages in cellular-level resolution and the ability to apply specific stains and immunohistochemical markers [2,46]. Optical imaging techniques including optical coherence tomography (OCT) and light sheet microscopy can achieve cellular-level resolution but are limited to small specimens or thin sections due to limited penetration depth [47]. Micro-CT provides excellent resolution for mineralized tissues but poor soft tissue contrast [48].

### 9.3. Limitations and Challenges

It is essential to acknowledge the substantial practical limitations inherent to HF-MRI that constrain its widespread application and throughput. These limitations—primarily centered on acquisition time, resource requirements, and accessibility—fundamentally shape the role of HF-MRI in developmental neuroscience and clinical research. They do not diminish the scientific value of the findings but provide essential context for interpreting results and planning future studies. High field MRI is a powerful but resource-intensive tool best deployed for questions requiring its unique capabilities, complemented by more accessible imaging approaches for broader applications.

#### 9.3.1. Acquisition Time Constraints

The most significant practical limitation is the extremely long acquisition time required for high-resolution imaging. The study achieving 27 µm resolution reported scan times of approximately 24 h per specimen [12]. This extended acquisition is necessary to accumulate sufficient signal averages to overcome thermal noise at such high resolution. The number of voxels increases as the cube of the linear resolution improvement; reducing voxel size from 100 µm to 27 µm increases the number of voxels by a factor of approximately 50, requiring proportionally more signal averaging to maintain SNR. The 11.7 T protocols developed for the p-HCP similarly employ extended scan times, with complete multimodal acquisitions requiring multiple days per specimen when including anatomical imaging, relaxometry, and high angular resolution diffusion imaging [1,6]. For larger specimens that exceed the scanner bore, blockwide acquisition and digital reconstruction are employed, further extending total acquisition time [6]. When the complete multimodal protocol is implemented, including T1-weighted imaging (50 μm, 8 h), T2*-weighted imaging (50 μm, 6 h), diffusion-weighted imaging (100 μm, 14 h), and quantitative relaxometry sequences (4–6 h), the total scan time approaches 48 h per specimen [1,6].

In practice, accounting for setup time, quality assurance scans, and necessary scanner maintenance, the actual calendar time extends to 3–4 months for a 15-specimen cohort. These time requirements impose severe constraints on study design. Longitudinal studies tracking developmental changes across multiple time points become logistically prohibitive. Case–control studies comparing pathological specimens to age-matched controls face significant delays in data acquisition. Time-sensitive studies—such as those requiring correlation with fresh tissue analyses or genetic studies with degradation-sensitive assays—may be incompatible with multi-day imaging protocols.

#### 9.3.2. Throughput and Productivity Limitations

The long acquisition times translate directly to severely limited throughput, constraining the scale and scope of feasible studies [12]. A single 11.7 T scanner operating at maximum capacity (allowing for 20% downtime for maintenance and setup) can image approximately 50–60 specimens per year with the full multimodal protocol, or 200–240 specimens per year with the primary T2-weighted sequence only.

This throughput limitation has several critical implications for research design and statistical power. First, achieving sample sizes adequate for robust statistical analysis of rare developmental variants or subtle pathological changes (typically N = 50–100 per group) would require 1–2 years of dedicated scanner time for a single study. Second, the limited throughput creates difficult prioritization decisions. A scanner capable of imaging 50 specimens annually must choose between depth (comprehensive multimodal characterization of fewer specimens) and breadth (single-contrast imaging of more specimens). Third, pilot studies and protocol optimization—essential components of rigorous experimental design—consume significant scanner time. This “overhead” further reduces effective throughput for hypothesis-driven research. Fourth, the low throughput creates vulnerability to technical failures. A single scanner malfunction requiring days or weeks for repair can compromise months of carefully planned experiments.

#### 9.3.3. Resource Requirements and Accessibility

High-field MRI demands extraordinary resource investments that limit accessibility to a small number of specialized centers. The limited number of suitable high-field centers worldwide and the substantial resource requirements at each site make such collaborations logistically and financially challenging.

The primary resource requirements include equipment costs, infrastructure requirements, operating costs, and personnel requirements. The long acquisition times and high costs underscore the value of comprehensive multimodal characterization when specimens are scanned. Resource intensity means high-field MRI is most appropriately deployed for questions requiring its unique capabilities, specifically, anatomical characterization at resolutions approaching histology. Applications not requiring sub-100 μm resolution may be more efficiently addressed with lower-field systems (3 T) offering faster acquisition and greater accessibility [6].

#### 9.3.4. Limitations of Ex Vivo Imaging

Several limitations inherent to ex vivo imaging must be acknowledged. (1) Fixation alters tissue properties, particularly T1 and T2 relaxation times, which are substantially shorter in fixed tissue compared to in vivo. While this does not affect anatomical measurements (structure volumes, cortical thickness), it means relaxometry values cannot be directly applied to in vivo imaging. However, the relative differences between structures and developmental trends remain informative. (2) Fixation may cause tissue shrinkage, typically 5–15% depending on fixation protocol and tissue type [49]. (3) Ex vivo imaging lacks functional information. While diffusion imaging reveals white matter organization, it does not capture active neuronal signaling or hemodynamic responses [50]. Complementary in vivo studies are needed to relate structural development to functional maturation. (4) The postmortem specimens may not represent normal development if the cause of death affected brain development. (5) The small-bore size of high-field preclinical MRI systems limits the size of specimens that can be imaged [6]. For larger fetal brains (>30 gestational weeks), blockwide acquisition and digital reconstruction are necessary, introducing potential registration errors and discontinuities [6]. This limitation is particularly relevant for studies spanning wide gestational age ranges [1].

#### 9.3.5. Sample Size and Statistical Power

The limited throughput dramatically limits the sample size and statistical power of studies. HF fetal brain cohorts remain modest throughout the studies. The limitations of HF-MRI constrain the number of specimens that can be imaged and may introduce selection bias if only the highest quality specimens are chosen for imaging [51]. Sex-specific and age-related developmental trajectories, respectively, rare anatomical variants or subtle regional differences may require larger cohorts for adequate statistical power. While correlations with histology provide validation, the specific cellular and molecular features that determine T1, T2, and diffusion properties in the developing brain are complex and may vary across developmental stages [52].

### 9.4. Reproducibility and Standardization

As high-resolution fetal brain imaging transitions from pioneering studies to more widespread application, issues of reproducibility and standardization become increasingly important. Differences in field strength, hardware, pulse sequences, specimen preparation, and image processing can all affect results, making cross-study comparisons challenging [53]. The development of standardized protocols, such as those employed in the p-HCP, represents an important step toward reproducible imaging [1,6]. Sharing of raw data, processing pipelines, and quality control metrics through open science initiatives will facilitate validation and extension of findings across research groups [54].

### 9.5. Ethical Considerations

Research involving human embryonic and fetal tissue raises important ethical considerations. All studies reviewed here obtained appropriate ethical approval and informed consent from donors [1,2,6,12]. The use of tissue from elective terminations or spontaneous pregnancy losses must be conducted with respect for donor autonomy and in accordance with local regulations and ethical guidelines [55]. The potential future application of high-resolution imaging to clinical assessment of fetal abnormalities raises additional ethical questions regarding prenatal diagnosis, parental decision-making, and the potential for discrimination based on fetal characteristics [56]. These issues require ongoing dialogue among researchers, clinicians, ethicists, and patient advocates to ensure that advances in imaging technology are applied in ways that respect human dignity and promote beneficial outcomes [57].

## 10. Future Directions and Clinical Translation

### 10.1. Advancing Toward Cellular-Level Resolution

Further advances toward microscopic resolution (1–10 µm) remain an important goal. At this resolution, individual neurons, glia, and vascular elements could be visualized, enabling direct comparison with histological preparations and potentially eliminating the need for destructive sampling [58]. Achieving microscopic resolution will require continued advances in multiple areas. Higher field strengths (14 T and above) are under development and may provide the SNR needed for single-digit micrometer resolution [59]. Improved gradient systems with strengths exceeding 1000 mT/m will enable shorter echo times and more efficient spatial encoding [60]. Advanced reconstruction techniques including compressed sensing, machine learning-based denoising, and super-resolution methods may extract additional information from acquired data [61].

### 10.2. Integration with Computational Modeling

The detailed anatomical and connectivity data provided by high-resolution MRI create opportunities for computational modeling of brain development. Finite element models of cortical folding can be constrained by empirical measurements of cortical thickness, surface area, and mechanical properties derived from MRI [62]. Network models of connectivity can be initialized with tractography-based connectivity matrices and used to simulate the emergence of functional networks [63]. Integration of imaging data with gene expression, cell lineage tracing, and other molecular data will enable multiscale models that link genetic programs to cellular behaviors to tissue-level organization [64]. Such models can generate testable predictions about developmental mechanisms and identify critical periods when perturbations are most likely to cause lasting deficits [65].

### 10.3. Clinical Translation: Challenges and Opportunities

While the high-resolution imaging described here is currently restricted to ex vivo research applications, there is considerable interest in translating these advances to clinical fetal imaging. In vivo fetal MRI at 3 T has made substantial progress in overcoming motion artifacts through fast acquisition sequences and motion correction algorithms [4,66]. Extension to 7 T for in vivo fetal imaging is under investigation, with early studies demonstrating feasibility and improved resolution compared to 3 T [67]. However, significant challenges remain for clinical translation of high-field fetal MRI. Safety considerations including SAR, acoustic noise, and potential effects on the developing fetus must be carefully evaluated [68]. Motion artifacts remain a major challenge, as fetal movement cannot be controlled and sedation is not feasible [5]. The limited bore size of current 7 T systems constrains positioning options for pregnant subjects [69]. Despite these challenges, incremental advances in clinical fetal MRI resolution, from the current 500–1000 µm to 300–500 µm, could significantly improve the detection of subtle abnormalities and enable earlier diagnosis of developmental disorders [70].

### 10.4. Applications for Neurodevelopmental Disorders

A major motivation for advancing fetal brain imaging is to identify early markers of neurodevelopmental disorders, including autism spectrum disorder, schizophrenia, cerebral palsy, and intellectual disability [71]. Many of these conditions have prenatal origins, with disruptions in neuronal migration, connectivity, or cortical organization occurring during fetal development [72]. High-resolution imaging of post-mortem tissue from affected individuals could reveal subtle anatomical abnormalities not visible at conventional resolution [73]. Comparison with normative atlases derived from typically developing specimens would enable quantitative assessment of deviations in cortical thickness, lamination, connectivity, and other features [30]. Integration with genetic data could identify genotype-phenotype relationships and elucidate pathogenic mechanisms [74]. Ultimately, the goal is to translate findings from ex vivo studies to in vivo biomarkers that can be assessed during pregnancy, enabling early intervention and potentially improving outcomes [75]. While this translation faces substantial technical and ethical challenges, the detailed anatomical and mechanistic understanding provided by high-resolution ex vivo imaging is an essential foundation for this effort [76].

### 10.5. Open Science and Data Sharing

The complexity and cost of high-resolution fetal brain imaging make data sharing particularly important. Individual research groups can image only limited numbers of specimens, but pooling data across groups can create large, diverse data sets that enable robust statistical analysis and generalization [77]. Several initiatives are promoting the open sharing of fetal brain imaging data. The p-HCP is making its 11.7 T multimodal imaging data publicly available, providing a valuable resource for the research community [1,6]. The Allen Brain Atlas provides integrated gene expression and anatomical data for the developing human brain [78]. The Developing Human Connectome Project provides high-quality in vivo and ex vivo imaging data spanning the perinatal period [79]. The creation of reference atlases based on large, diverse samples will provide benchmarks for evaluating new studies and identifying deviations from typical development [29,30]. Continued expansion of these resources, along with development of standardized data formats, quality control procedures, and analysis tools, will accelerate progress in understanding brain development and translating findings to clinical applications [80,81].

## 11. Conclusions

The achievement of HF-MRI of human embryonic and fetal brain represents a landmark advance in developmental neuroimaging. The integration of high-resolution anatomical imaging with quantitative relaxometry, diffusion MRI, and histological validation provides comprehensive characterization of brain development. These advances offer transformative potential for understanding normal brain development, creating high-resolution developmental atlases, identifying early markers of neurodevelopmental disorders, and ultimately improving clinical assessment and intervention. While significant challenges remain—including the restriction to ex vivo imaging, extended acquisition times, and the need for continued validation—the trajectory of progress is clear. Continued advances in hardware, pulse sequences, and reconstruction methods promise to push resolution toward the cellular level, while efforts to translate findings to clinical in vivo imaging may enable earlier detection of developmental abnormalities. As imaging capabilities continue to advance and are integrated with molecular, genetic, and computational approaches, our understanding of how the human brain develops—and how development can go awry—will deepen substantially.

## Figures and Tables

**Table 1:** MRI Resolution Scales and Biological Structures.

Resolution Scale	Voxel Size Range (μm)	Biological Structures Resolved	Imaging Modalities
Microscopic	<10	Individual neurons (10–20 μm), glia (5–15 μm), capillaries (5–10 μm), dendrites, synapses	Light microscopy Electron microscopy Confocal microscopy
Mesoscopic (High)	10–30	Cortical layers (50–500 μm), small nuclei (100–500 μm), transient zones (100–500 μm), white matter fascicles (50–200 μm)	HF-MRI (ex vivo) μCT * OCT ^§^
Mesoscopic (Mid)	30–60	Cortical layers, medium nuclei, major white matter tracts	HF-MRI (ex vivo) 7 T MRI
Mesoscopic (Low)	60–100	Large nuclei, major structures, thick cortical layers	HF-MRI 7 T MRI
Submillimeter (High)	100–300	Major structures, sulci/gyri, large tracts, ventricles	7 T MRI (in vivo) 3 T MRI
Submillimeter (Mid)	300–600	Major structures, gross anatomy	3 T MRI (research)
Submillimeter (Low)	600–1000	Major structures only	3 T MRI (clinical fetal)
Macroscopic	>1 mm	Lobes, large ventricles, gross landmarks	Clinical MRI/CT

* μCT: micro–Computerized Tomography; ^§^ OCT: Optical Coherence Tomography.

**Table 2:** Comparative Analysis of High Resolution Embryonic and Fetal Brain MRI Studies *. * All specimens were investigated ex vivo. ^§^ Carnegie stage.

Reference	Field Strength	Resolution (µm^3^)	Gestational Age (Weeks)	Key Structures Visualized
Wang et al., 2015 [2]	4.7 T	130–200	10–18	Cortical layers, germinal zones
Arcamone et al., 2025 [6]	11.7 T	100–200	18, 27, 31	Whole brain anatomy connectivity, relaxometry
Makihara et al., 2023 [10]	9.4 T	10–12	CS ^§^ 16	Whole embryo microstructure
Kunieda et al., 2024 [11]	9.4 T	30	CS ^§^ 23	Cortical layers accessory nerves
Boitor-Borza et al., 2021 [12]	7.04 T	27	9–14	Ganglionic eminences
Huang et al., 2013 [13]	11.7 T	200–600	13–16	Cortical microstructure, DTI

## Data Availability

No new data were created or analyzed in this study.

## Abbreviations

The following abbreviations are used in this manuscript:

HF-MRI	high-field magnetic resonance imaging
SNR	signal-to-noise ratio
RF	radiofrequency
SAR	specific absorption rate
FISP	Fast imaging with steady-state precession
RARE	relaxation enhancement
TSE	turbo spin echo
p-HCP	premature Human Connectome Project
FA	fractional anisotropy
DTI	diffusion tensor imaging
HARDI	high angular resolution diffusion imaging
